# The Hospital Isolation of Infectious Diseases

**Published:** 1913-07-19

**Authors:** H. Franklin Parsons

**Affiliations:** Late of the Medical Department, Local Government Board.


### THE HOSPITAL ISOLATION OF INFECTIOUS DISEASES.*

### Some Arguments for and against Isolation.

By H. FRANKLIN PARSONS, M.D., D.P.H., late of the Medical Department, Local Government Board.

The useful purposes aimed at by the removal to hospital of cases of infectious disease are of threefold sort, viz.:

1. The benefit of the individual patient by giving him better accommodation, food, and attendance than he might be able to obtain at his home, thus relieving his sufferings and promoting his recovery.

2. The protection of the health of other members of the household and of the general community by the removal and isolation of persons capable of imparting infection.

3. The obviating of the inconveniences and pecuniary losses entailed by the presence in a household of a case of infectious illness.

But while these advantages are undeniable, it must be admitted that the hospital isolation of infectious diseases has not entirely fulfilled the hopes which were entertained by the more sanguine of its advocates, and that it has been found to present certain incidental dangers and disadvantages. Its usefulness in arresting an outbreak at an early stage, if the first cases can be got hold of, is more obvious than its effect in extinguishing a developed epidemic or in reducing the prevalence of a disease endemically present. Among the objections which have been raised in recent years to the hospital isolation of patients with infectious diseases are the following:

1. That it cannot be shown to have diminished the prevalence of these diseases.

2. That the aggregation of cases produces a more virulent "hospitalised" strain of infection.

3. That the mortality is higher among cases treated in hospital than among those treated at home.

4. The danger to patients admitted to hospital under a wrong diagnosis.

5. The danger of cross-infection—*i.e.* of contracting a second disease from other patients in hospital.

6. The greater persistence of infectiousness in patients treated in hospital, so that they give rise to other cases ("return cases") on their return home.

7. The great cost of erection and maintenance of isolation hospitals; the money, it is said, might be spent in other ways with greater benefit to the public health.

8. Hospital isolation, it is said, is unnecessary, as infectious disease can be prevented from spreading by other means—*e.g.*, by Dr. Milne's treatment of scarlet fever by inunction of eucalyptus oil and swabbing the throat.

The above objections in the main have arisen out of, and apply to, the hospital treatment of scarlet fever, a disease which, while it forms the bulk of the cases admitted to hospital, is now a comparatively mild affection with a case-mortality, as shown by the table given in the previous article, of barely 2 per cent. Enteric fever and diphtheria ("clinical"), the other diseases now commonly treated in isolation hospitals, are dangerous maladies needing skilled attendance and careful nursing, and obviously require hospital treatment, either in an isolation or a general hospital, for the patient's own sake. The more formidable epidemic diseases for which isolation hospitals were originally provided have, as a matter of fact, been practically "stamped out," at any rate as regards their endemic prevalence.

The following table shows the annual mortality in England and Wales, per million persons living, from the principal infectious diseases during the period 1881-1912:—

Table II.

| Period | | Scarlet Fever | Enteric Fever | Diphtheria | Small-pox | Measles | Whooping-Cough |
|---|---|---|---|---|---|---|---|
| 5 years | 1881-5 | 436 | 216 | 156 | 78 | 413 | 459 |
| ,, | 1886-90 | 241 | 179 | 170 | 14 | 468 | 444 |
| ,, | 1891-5 | 182 | 144 | 253 | 20 | 407 | 398 |
| ,, | 1896-1900 | 135 | 175 | 272 | 7 | 421 | 359 |
| ,, | 1901-5 | 126 | 113 | 204 | 25 | 326 | 300 |
| ,, | 1906-10 | 86 | 69 | 154 | 0.2 | 291 | 253 |
| Year | 1911 | 50 | 70 | 130 | 0 | 360 | 210 |
| ,, | 1912 | 50 | 40 | 110 | 0 | 350 | 230 |

It will be seen from this table that during the period in which hospital isolation has come into vogue there has been on the whole a great reduction in the death-rates from the infectious diseases ordinarily treated in hospital, while in the death-rates from measles and whooping-cough, infectious diseases which are not ordinarily received into isolation hospitals, the reduction has been of much less extent. It would, however, be too much to assert that the reduction in the death-rates from infectious diseases is wholly or chiefly the effect of hospital isolation. During the above period the general sanitary condition and administration of the kingdom have greatly improved; and other preventive measures besides hospital isolation have doubtless had a large share. Thus the provision of pure water-supplies, the substitution of water-closets for privies, and the supervision of shellfish beds have undoubtedly had much to do with the diminution of enteric fever, while the decline in the mortality from diphtheria is concurrent with the coming into use of the methods of bacteriological diagnosis and antitoxin treatment. Moreover, epidemiologists know that infectious diseases vary greatly in virulence and fatality from time to time without apparent cause, although the fact that all these diseases have concurrently diminished may give sanitarians ground for claiming that their

* The first article appeared on June 21.

efforts, including hospital isolation, have had some share in the result.

It may be remarked, too, that this diminution has taken place in the face of a progressive urbanisation of the population of the kingdom, a process which by itself would have tended in the opposite direction, as the death-rates from these diseases are higher in urban than in rural populations. The following table shows the death-rates per million inhabitants in 1912 from the above diseases respectively in 95 great towns with a population exceeding 50,000 each (including London), in 146 smaller towns with populations ranging from 20,000 to 50,000, and in the remainder of England and Wales—*i.e.* in small towns and rural districts:—

Table III.

| Area | Scarlet Fever | Enteric Fever | Diphtheria | Small-pox | Measles | Whooping-Cough |
|---|---|---|---|---|---|---|
| 95 Great towns | 60 | 40 | 130 | 0 | 470 | 260 |
| 146 Smaller ,, | 60 | 50 | 110 | 0 | 350 | 240 |
| Rest of England and Wales | 40 | 40 | 100 | 0 | 200 | 170 |

Another factor, operative during the last thirty to forty years, which may be thought of as tending to render infectious diseases more prevalent, is increased school attendance; these diseases being very frequently contracted by children at school, especially at public elementary schools. School attendance was made compulsory in this country in 1870; its enforcement was, however, at first far from complete, especially in rural districts, but as years go on it has been more and more stringent. Sir Shirley Murphy has shown how the weekly number of cases of infectious diseases in London drops when the schools are closed for the holidays, and mounts rapidly when they are reopened.

### Criticisms of Statistics.

Considering, now, the objections to hospital isolation in so far as they apply especially to scarlet fever, we notice that during the period in which isolation hospitals have been coming into general use the death-rate from scarlet fever and the case-mortality have shown a notable diminution. The critics, however, aver that the explanation of this diminution is to be found in a changed type of the disease; and they point out that it has occurred in places where hospital isolation is not practised, and in other countries besides the British Isles: it is, in fact, a world-wide phenomenon of which the cause is not known. It cannot be shown, they say, that any marked reduction of the number of cases occurring has followed the establishment of isolation hospitals, however far their use may be pushed, or that towns which do not isolate scarlet fever, or do so only in a small proportion of cases, suffer a larger prevalence of the disease than those where the policy is to get every case possible into hospital.

There are, however, considerable difficulties in applying a statistical test to the question; these arising out of the circumstance that scarlet fever, like other infectious diseases affecting children, tends to recur in epidemic form at intervals of a few years, when a stock of subjects unprotected by a previous attack has accumulated. Indications of this tendency may be seen in the figures for London given in the table in the first article; and it is more obvious in those for smaller and more compact and homogeneous towns. Now, just as it may make a million pounds' difference in the receipts of the Chancellor of the Exchequer whether the financial year happens to include two Easters or none, so it will make all the difference to the figures of prevalence of scarlet fever in a district whether the period taken includes one or more epidemics or an inter-epidemic interval only. By taking sufficiently long periods for comparison we may be able to avoid this difficulty, but then the question of change of type comes in, scarlet fever showing more tendency to spread at some times than at others.

If, instead of comparing the figures of the same district at different periods, we compare those of different districts at the same period, similar difficulties are met with. The period taken may include an epidemic in one district and an interval only in the other. There are also differences between different districts, as regards such matters as age-constitution, and social position of the inhabitants, and housing conditions, which may affect their liability to prevalence of scarlet fever. The districts which habitually suffered most ravages from scarlet fever might be expected to be the first to see the need for an isolation hospital.

### Hospital and Home Treatment Compared.

It is also objected that the proportion of secondary cases per household has not been less in households where the first case has been removed to hospital than in those where it was treated at home. It must be remembered, however, that removal to hospital would be more likely to take place from households in which there were other susceptible persons or in which facilities for isolation were wanting, than from those where contrary conditions existed. Whether the infection giving rise to secondary cases be derived from the first case prior to removal, from infected articles or places which had not been properly disinfected, or from prolonged infectiousness of the patient after release from isolation, it is obvious that the number of secondary cases must depend on the number of susceptible persons in the household. It has been shown by Dr. Malet at Wolverhampton and by Dr. Arnold at Manchester that when the comparison between hospital treatment and home treatment is based, not on the proportion of secondary cases to invaded households, but on the proportion to the number of susceptible persons in those households, there is a considerable balance of advantage in favour of removal to hospital, notwithstanding the occurrence of a certain number of "return cases." The following reasons, in addition to the increasing urbanisation and more general school attendance already mentioned, seem sufficient to account for the effect of hospital isolation upon the prevalence of scarlet fever not having been so great or obvious as was at one time expected.

1. When the disease is of so mild a type as at present there are many slight cases which escape detection, and these going about among other people are more likely to spread the disease outside the household than the severer cases which are confined to bed.

2. The removal of the first case to hospital usually does not take place until there has been time for other persons to contract the disease. The doctor is perhaps not called in until the patient has been ill a day or two; if he finds the case to be scarlet fever he probably waits till the evening when he has finished his rounds to post the notification certificate to the health officer, who receives it next day. Even then the case may have to be visited, and it may not be possible to arrange for removal the same day. As scarlet fever is infectious from the commencement of symptoms, there may thus be several days during which the patient may infect other members of the household before his removal to hospital.

3. A scarlet fever patient is apt to remain infectious for a considerable but uncertain time after his apparent recovery, and on his return from hospital may give rise to other cases ("return cases") among susceptible members of his household. The same thing may happen when a patient who has been treated at home is released from isolation; but it is thought that persistent infectiousness is commoner among hospital-treated than among home-treated patients; and in the present state of knowledge there is no certain means of determining that a patient is free from infection, and the occurrence of occasional return cases cannot be altogether prevented however long the patient may be kept in hospital. Indeed, prolonged detention seems to have no effect in reducing their number. The proportion of return cases is small, usually between two and four per cent. of the cases discharged; but their occurrence has tended more than anything else to throw doubt on the utility of hospital isolation.

4. Outbreaks of scarlet fever occur from time to time from causes other than direct personal infection—*e.g.* through infected milk. Thus a supply of milk infected by a case of scarlet fever in the family of a dairyman in a rural district in which there is no isolation hospital may be the cause of a hundred cases in a distant town well provided with hospital accommodation.

### The Risks of Hospital Treatment.

As regards the allegation that the aggregation of cases of scarlet fever produces a more virulent strain of infection, it is possible that this may be so in overcrowded and ill-kept hospitals, but the great reduction in case-mortality shown in Table I. precludes the idea that there has been any general increase of virulence as the result of the treatment of the disease in properly managed hospitals. Nor can this opinion be supported by the fact that the case-mortality, as shown in the table, is somewhat higher among the cases removed to hospital than among the notified cases generally, for the reason that the comparative case-mortality of hospital and home-treated cases is essentially dependent on the initial severity of the cases as determining removal. If the severe cases are removed to hospital and the mild cases are left at home the mortality among hospital cases must be expected to be the greater, apart from any unfavourable influence of the hospital or of the journey. On the other hand in more dangerous diseases if the worst cases could not bear removal or died before it could be attempted, and only the less severe ones were taken to hospital, the hospital cases would show a less mortality than the home-treated ones. In the earlier years, in Table I., when less than half of the notified cases of scarlet fever in London were taken to hospital, the case-mortality in hospital was considerably higher than among notified cases generally. There was then an insufficiency of accommodation, and probably preference was given to the severest and most urgent cases; but as the proportion of removals increased the difference in the case-mortality diminished. The proportion of deaths among the small number of notified cases (now about 10 per cent.) not removed to hospital is very low; but these would include, not only mild cases and cases among persons in good circumstances, but also duplicate notifications and missed cases only discovered during convalescence.

The risk run by patients admitted to hospital under an erroneous diagnosis, the danger of cross-infection in hospital, and the liability to the occurrence of return cases already mentioned are, it must be admitted, drawbacks to the usefulness of hospital isolation, and every care should be taken to guard against them as far as possible; but their magnitude is not sufficient to counterbalance the advantages of hospital isolation where the patient has not sufficient facilities for isolation and treatment at home.

The cost of the erection and maintenance of isolation hospitals is no doubt considerable; and care should be taken to keep it down by combination in the case of the smaller districts, by avoiding superfluities and extravagance in building and by careful management. But assuming that the expenditure is no greater than the protection of the public and the welfare of the patients require, the cost of maintaining isolation hospitals, like that of fire-stations and other defences, may be regarded as in the nature of an insurance against the dangers and losses caused by infectious diseases.

The alternative methods which have been suggested as substitutes for isolation hospitals do not fulfil the same purposes, or are not applicable in all cases, especially where the patient has not proper lodging and accommodation at his home. Thus Dr. Milne claims that by his method of treatment he has been able, without isolation, to prevent the spread of scarlet fever among the children in Dr. Barnardo's Homes, but it has not been shown that elsewhere than in such an institution this treatment could be applied at the very commencement of the illness or carried out with the frequency which Dr. Milne deems necessary.

To sum up the reasons for and against the hospital isolation of scarlet fever. There are a certain number of cases in which the patient has not proper lodging and accommodation or the necessary attendance which his state requires; for these removal to hospital is necessary. In a larger number he has not accommodation or attendance so good as he would get in a hospital, or if there is no serious deficiency from the point of view of his own welfare, his isolation may be impossible, and he may be nursed perhaps by the mother of the family who has other household duties to perform; in such cases removal to hospital is generally advisable. Or the case may occur under circumstances involving special danger or alarm to the public, as at a dairy farm or milk-shop, or at a post-office or other frequented place of business; or its presence may involve serious inconvenience or pecuniary loss, as at a boarding school or an hotel; in such cases removal to hospital is necessary. In the days before isolation hospitals were provided, cruel cases used from time to time to occur in which a servant ill of scarlet fever was sent by a long road journey to her home in another district, thus introducing the disease there, perhaps into a crowded cottage full of children. Nothing could be done to prevent this practice, as the mere act of removal was not illegal if there was no wilful exposure of the infected person; and the employer, if remonstrated with, could reply that the safety of his household required that patient's removal, and there was no other place to send her to except her home.

On the other hand, patients of the well-to-do class, for whom proper accommodation, attendance, and means of isolation are available at home, escape by home treatment certain dangers which they would incur if sent to a hospital, though removal to a hospital may sometimes be preferred on grounds of convenience or expense. The removal of the first case to hospital will often prevent any further spread of the disease; but when an epidemic has become developed it is inadvisable to press removal to the extent of overcrowding the hospital.

Hence while hospital treatment of scarlet fever is necessary in a certain number of cases, and unnecessary in others, the extent to which it is advisable to carry it will vary with local circumstances, such as the housing and social condition and occupation of the inhabitants, and more especially whether they get their living in ways which would be interfered with by the presence of infectious disease in their households. There is room, therefore, for legitimate differences of opinion among medical officers of health, and of practice in different districts, in respect to the extent to which hospital isolation of scarlet fever should be practised. The following are the views of Dr. Millard, of Leicester, a well-known critic of hospital isolation (*Public Health*, May, 1902):—

"That, whilst the provision of hospital isolation for persons suffering from scarlet fever is a very great convenience to the public, it is doubtful, from the point of view of controlling the disease, whether the results achieved by the wholesale removal of cases to hospital are commensurate with the heavy cost entailed. It is possible that results equally satisfactory would be obtained by the removal to hospital of the more urgent cases only.

"Also that in no case is the wholesale removal to hospital of scarlet fever cases to be recommended if it involves overcrowding of the available hospital accommodation."

These views by no means negative the usefulness of hospital isolation in appropriate cases.

